# Prophylactic Onlay Mesh Implantation During Definitive Fascial Closure After Open Abdomen Therapy (PROMOAT): Absorbable or Non-absorbable? Methodical Description and Results of a Feasibility Study

**DOI:** 10.3389/fsurg.2020.578565

**Published:** 2020-12-15

**Authors:** Sebastian Schaaf, Robert Schwab, Christoph Güsgen, Arnulf Willms

**Affiliations:** Department of General, Visceral and Thoracic Surgery, German Armed Forces Central Hospital Koblenz, Koblenz, Germany

**Keywords:** open abdomen, prophylactic mesh, incisional hernia, onlay, laparotomy

## Abstract

**Introduction:** Incisional hernia development after open abdomen therapy (OAT) remains a common complication in the long run. To demonstrate the feasibility, we describe our method of prophylactic onlay mesh implantation with definitive fascial closure after open abdomen therapy (PROMOAT). To display the feasibility of this concept, we evaluated the short-term outcome after absorbable and non-absorbable synthetic mesh implantation as prophylactic onlay.

**Material and Methods:** Ten patients were prospectively enrolled, and prophylactic onlay mesh (long-term absorbable or non-absorbable) was implanted at the definitive fascial closure operation. The cohort was followed up with a special focus on incisional hernia development and complications.

**Results:** OAT duration was 21.0 ± 12.6 days (95% CI: 16.9–25.1). Definitive fascial closure was achieved in all cases. No incisional hernias were present during a follow-up interval of 12.4 ± 10.8 months (range 1–30 months). Two seromas and one infected hematoma occurred. The outcome did not differ between mesh types.

**Conclusion:** The prophylactic onlay mesh implantation of alloplastic, long-term absorbable, or non-absorbable meshes in OAT showed promising results and only a few complications that were of minor concern. Incisional hernias did not occur during follow-up. To validate the feasibility and safety of prophylactic onlay mesh implantation long-term data and large-scaled prospective trials are needed to give recommendations on prophylactic onlay mesh implantation after OAT.

## Introduction

Open abdomen therapy (OAT) is defined as the deliberate decision not to close the fascia at the end of laparotomy (1). This treatment strategy is an established cornerstone in the surgical management of critically ill patients with intraabdominal pathologies to reduce surgical traumatization. It has been shown that OAT reduces morbidity and mortality in patients with depleted systemic resources due to severe abdominal trauma or gastrointestinal disease (2).

The primary treatment goal is the sequential control of infectious or traumatic foci. Secondarily, the key issues are swift fascial closure and the prevention of enteroatmospheric fistulas (1). Vacuum-assisted wound closure nd mesh-mediated fascial traction (VAWCM) and other OAT techniques, which combine the synergistic effects of negative pressure wound therapy and dynamic fascial traction are the best available options for OAT nowadays (3). However, repetitive abdominal surgeries are necessary, which results in reasonable cumulative traumatization of the abdominal wall.

Incisional hernias are common complications of abdominal surgery with a reported incidence of at least 3–20% after laparotomies (4). Little is published on the specific aspects of incisional hernia development after OAT; however some monocentric retrospective studies showed the incisional hernia incidence after OAT to be far higher (35–66%) than after regular laparotomies (5–9). The development of incisional hernias depends on various factors such as surgical technique (e.g., incision type, suture technique, and material) or comorbidity (i.e., aortic aneurysm, obesity) (10, 11).

Incisional hernia development is associated with an impaired outcome, as the functional properties of the abdominal wall are altered, incarceration and emergency surgeries are omnipresent risks, and pain is a frequent symptom (12). Research data showed the reduced quality of life (SF36 questionnaire) in patients with an incisional hernia after OAT (5). Moreover, hernia repair itself comes with remarkable perioperative risks, especially if complex abdominal wall reconstruction becomes necessary due to giant hernias with an intestinal loss of domain condition (13).

Prophylactic mesh implantation is shown to be beneficial in high-risk patients with midline laparotomies (14, 15). Risk factors in that context are considered to be either patient-specific or surgery-relates. The former ones include factors such as obesity, connective tissue disorders or aortic aneurysms, diabetes, smoking, and corticosteroid medication (16). The most relevant factor associated with the surgical procedure itself is the actual technique of how fascial closure is obtained. The European Hernia Society has given recommendations on fascial closure, which involve the use of long-term absorbable sutures and a suture length to wound length (SL:WL) ratio of at least 4:1 (9, 11, 17).

A remarkable amount of evidence on prophylactic mesh implantation in high-risk patients after laparotomies has been grown (14, 17). Borab et al., for example, reported a reduction of incisional hernia risk of 85% (15). However, this comes at the cost of a higher seroma rate. These results were recently confirmed for emergency laparotomies, as well (16). Put these findings together; it seems reasonable to suppose that the fascial closure after OAT is a similar high-risk situation, both in terms of patient-specific or surgical-technical factors (11).

Currently, there is no evidence on prophylactic mesh implantation during delayed primary fascial closure operation after OAT. To display the feasibility of prophylactic onlay mesh implantation after OAT (PROMOAT), we evaluated the short-term outcome after absorbable and non-absorbable synthetic mesh implantation as prophylactic onlay.

## Materials and Methods

### Description of Surgical Technique

**Figure 1:**
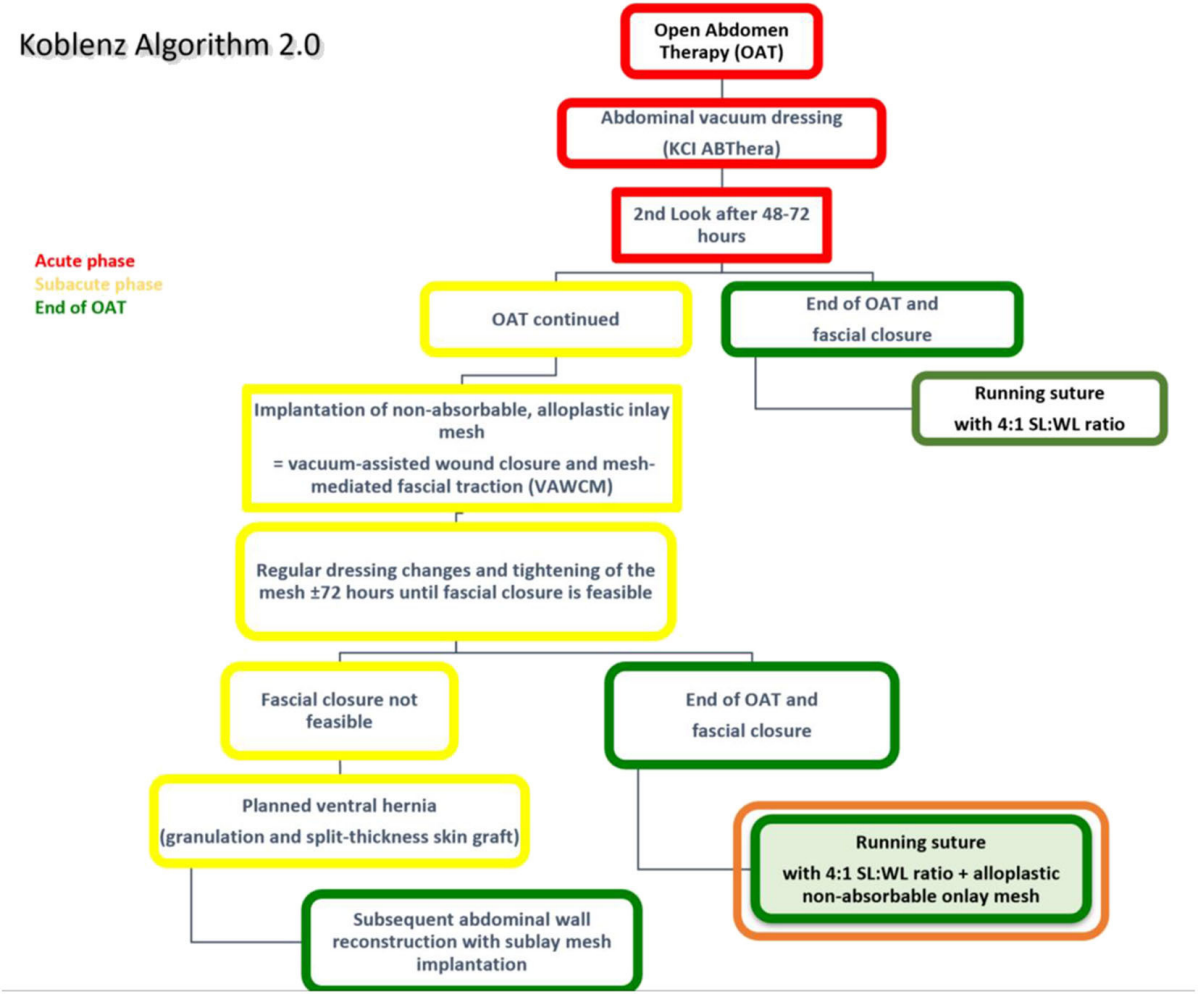
The amended Koblenz Algorithm (2.0) with special focus on the prophylactic onlay mesh implantation at the end of OAT.

The original technique of OAT (Koblenz Algorithm) has been described in detail previously (18). In this study, we present an amended method as a prophylactic onlay mesh is implanted at the delayed primary fascial closure operation (Koblenz Algorithm 2.0, Figure 1).

Patients with the indication for OAT and in whom primary fascial closure is impossible during the abdominal surgery are treated with a commercially available OAT dressing kit (ABThera^TM^ SensaTRAC^TM^ Open Abdomen Dressing, KCI Medical/3M, Maplewood, MN, United States). To protect the viscera from serosal lesions and prevent enteroatmospheric fistulas, a visceral protective layer integrated into the abdominal vacuum foam is implanted (19). It is placed deep laterally in the paracolic spaces to prevent lateral adhesions.

A scheduled second-look operation after 48–72 h is performed, and the decision is made whether it is possible to close the abdomen or to continue OAT. This initial period was considered the acute phase; hence in the former case, the abdominal fascia is closed following the recommendations by the EHS (17) but without a prophylactic onlay mesh. If the OAT has to be continued, an alloplastic non-resorbable mesh is sutured in inlay position to the fascial edges to achieve mesh-mediated fascial traction (VAWCM). The mesh is divided in the midline, and each half is sutured to the fascial edges with a resorbable running suture until it was sutured in the midline maintaining continuous moderate traction of the fascia. In the next step, another vacuum foam is cut to the size of the laparostomy and placed on the mesh. Then, the wound is closed with adhesive foil, and the suction is applied. Usually, a negative pressure of 75–100 mmHg is reasonable, but in special conditions (i.e., impaired coagulation), this is reduced to 25 mmHg.

During the next operation, the mesh is was re-opened in the midline, and the surgical revision is obtained. Depending on the intrabdominal pressure and swelling of the intestines, the fascial dehiscence is reduced by suturing the mesh tighter in the midline. This leads to continuous and progressive fascial traction and hence facilitates the delayed primary fascial closure.

As soon as it is considered possible, the mesh is removed, and the abdominal fascia is closed with a slowly absorbable running suture (Monomax®, poly-4-hydroxybutyrate, B. Braun, Melsungen, Germany) following EHS guidelines of the abdominal wall closure (17). This condition is defined as definitive fascia closure, i.e., the complete closure of the fascia edges with no remaining fascial gap (fascia-to-fascia closure) and is a pre-requisite for onlay mesh augmentation and inclusion in this study.

To prepare the abdominal wall for onlay mesh implantation, a sufficient dissection is done to warrant an epifascial overlap of at least 5 cm in all directions from the fascia-to-fascia closure. Either an alloplastic long-term absorbable mesh (TIGR®Matrix, Novus Scientific, Uppsala, Sweden) or an alloplastic non-absorbable mesh (Dynamesh CICAT, Dahlhausen, Aachen, Germany) is used for augmentation in onlay position in this study cohort. The reason for using two different mesh types was to check for feasibility in the OAT setting, and not to compare the outcomes. As the implantation of alloplastic material in patients with the history of peritonitis seemed potentially risky, we chose a two-step approach. Initially, the long-term absorbable mesh was implanted. After we observed no complications requiring invasive treatment, we also tried the implantation of the non-absorbable mesh because there is recent evidence that the risk of mesh infection and the need for explantation depends on the specific mesh material (20).

**Figure 2:**
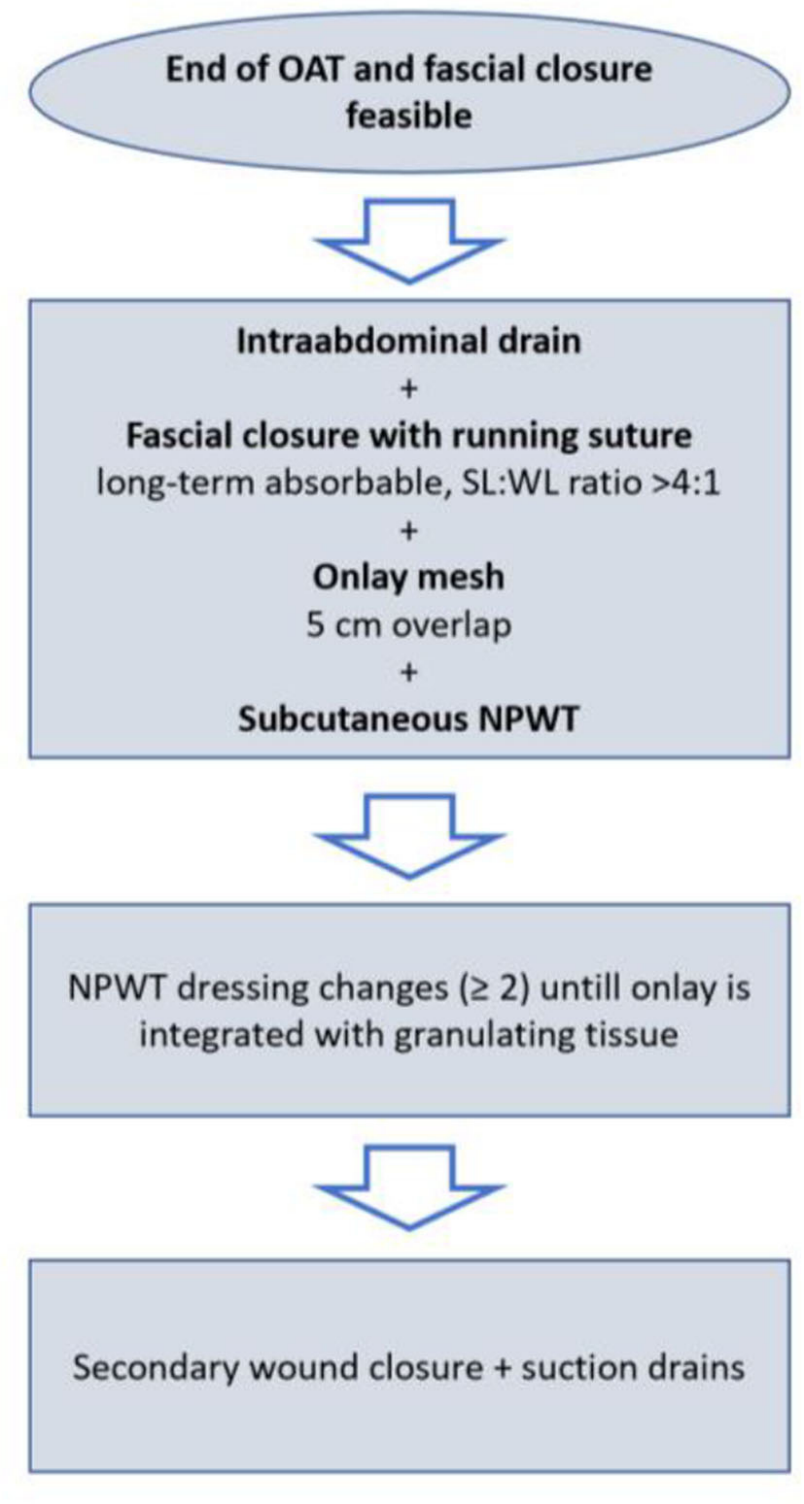
Detailed procedure of delayed primary fascial closure and prophylactic onlay mesh implantation.

**Figure 3:**
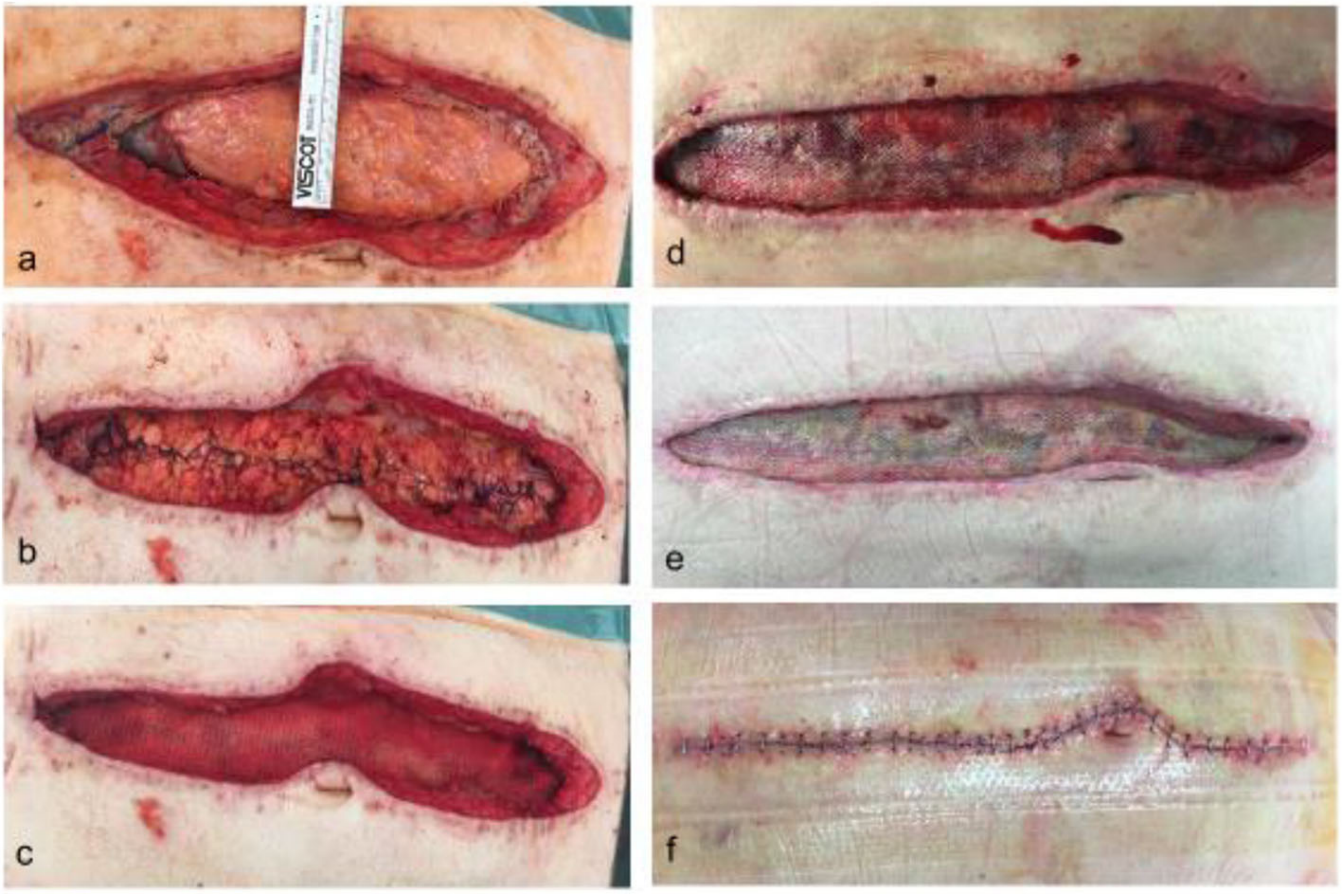
Post-operative course after definitive fascial closure with prophylactic onlay mesh. Picture a shows the residual fascial dehiscence at the of 15 days of OAT in a 75 yo male **(a)**. The indication for OAT was ACS following massive bleeding and transfusion. The fascial defect was sutured with a long-term absorbable running suture maintaining a suture length to wound length ratio of at least 4:1 **(b)**. After dissection of a proper epifascial space, a 30 ×10 cm long-term resorbable mesh was placed in onlay position and fixated with a non-absorbable running suture and a subcutaneous vacuum dressing was applied **(c)**. Intraabdominal drains had been placed previously. Picture **(d)** shows the situs at day 4 after definitve fascial closure with clean conditions and initial integration of the mesh by granulating tissue. On day 8 the mesh and the wound was well granulated, hence secondary wound closure was performed **(e)**. Lastly, **(f)** shows the wound 20 days after definitive fascial closure and end of OAT.

The mesh is fixed to the fascial tissue underneath with an absorbable running suture (Vicryl, polyglactin, B. Braun, Melsungen, Germany) and a negative pressure wound therapy (NPWT) is applied with continuous suction of 100 mmHg. NPWT dressings are changed at least two times until the onlay mesh is sufficiently integrated with granulating tissue, as we assume the mesh-associated seroma/hematoma risk to be lower. Afterwards, secondary wound closure with the placement of suction drains is performed (Figure 2). In particular, secondary wound closure is obtained in two layers with a subepidermal slowly absorbable suture and non-absorbable single epidermal stitches after subtle excision of the dermal wound edges. Additionally, Figure 3 shows the post-operative course after definitive fascial closure.

### Patient Population and Study Design

Patients have been prospectively included in this study.

**Figure 4:**
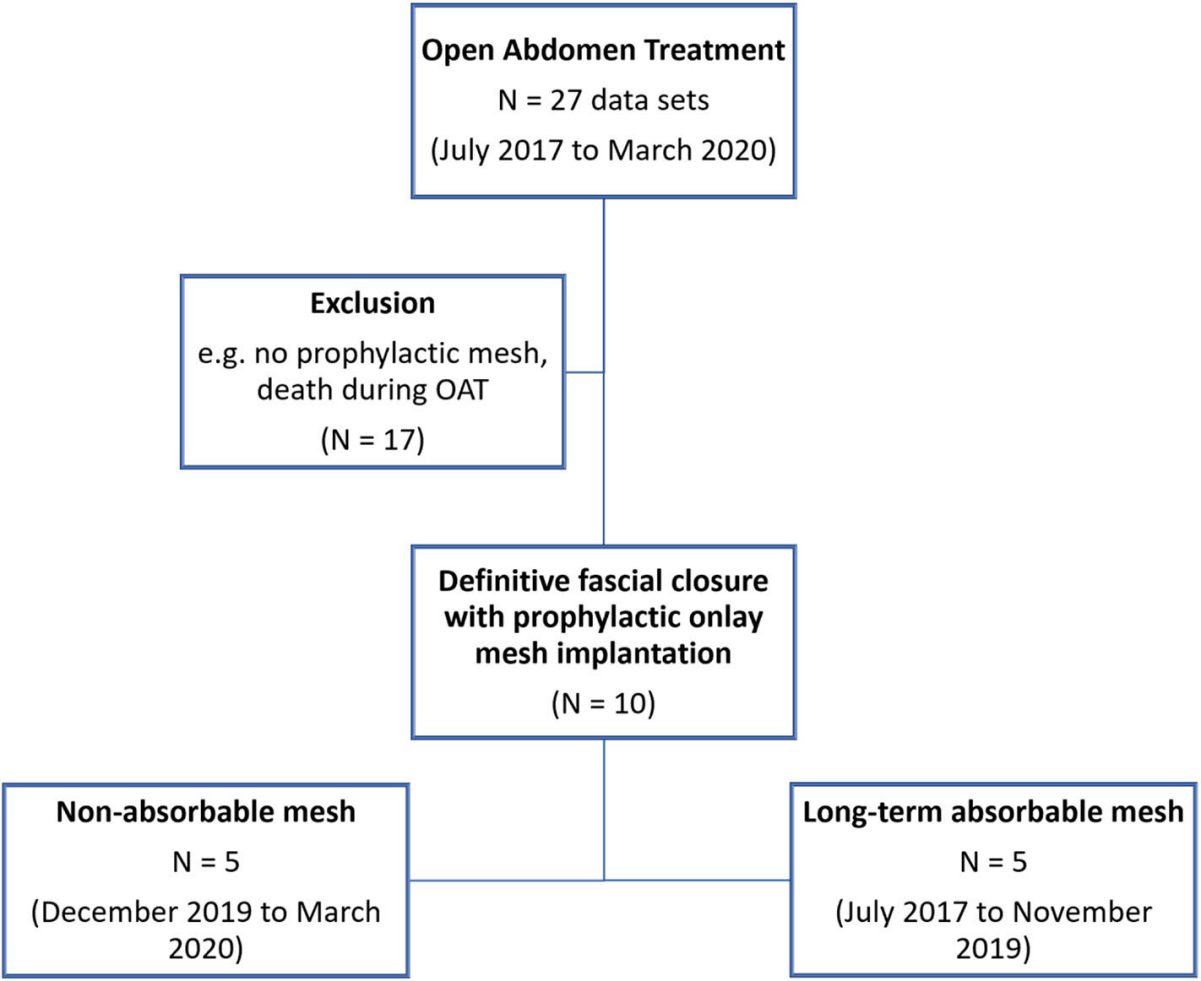
Flow chart of patient inclusion in the study.

Inclusion criteria were as follows:

- OAT at our facility between July 2017 and March 2020 (Figure 4)
- Definitive fascial closure (no remaining fascial gap) was possible, and a prophylactic onlay mesh has been implanted (PROMOAT)

Patients were excluded due to these reasons if the prophylactic onlay mesh implantation has been considered unfeasible:

- Surgeon's individual decision
- Expected survival was less than half a year
- End of OAT and definitive fascial closure was possible yet at the second look operation (acute phase of Koblenz Algorithm)
- Further abdominal surgery (e.g., ostomy reversal) was scheduled

The primary endpoint of this study was the occurrence of an incisional hernia during follow-up. Secondary endpoints were post-operative complications like seromas, hematomas, bleeding, burst abdomen, surgical site infections (SSI), and any complication with the indication for a redo surgery. Surgical site infections were defined by the CDC criteria (21). In this study, every CDC type of SSI (superficial, deep, and organ space) was considered a SSI. Complications have been classified following Clavien and Dindo (22). Invasive treatment of a complication was considered Clavien-Dindo grade III or higher, whereas non-invasive actions that had to be taken were grades I and II.

Patients' age, sex, and BMI, as well as the underlying disease and current surgical history, have been retrieved from the charts. Furthermore, surgery-related data, e.g., remaining dehiscence/fascial gap length and width, type of mesh, size, and fixation, have been documented. Lastly, the post-operative pain was rated with the numerical rating scale (NRS), 0 no pain; 10 worst pain). This scale is a simple 11-item scale that is commonly used as a pain assessment tool in the clinical routine and research (23). The patients were asked to rate their level of pain on a scale of 0 to 10.

### Data Management and Statistical Analysis

The collected data has been stored after pseudonymization. Informed consent has been obtained from the patients or their legal representatives. The local ethics committee approved this study (No. 2020-14884 of 25 March 2020).

The data analysis has been done with Excel (Excel 2016, Microsoft Corp., Redmont, United States) and SPSS (SPSS Statistics 20, IBM, Armonk, United States). Descriptive statistics have been calculated. Metric data is given in means ± standard deviation and 95% confidence interval. Categorical data are reported as proportions (percentages).Due to the low n, we assumed the data not to be normally distributed. Therefore, differences between groups were tested with either contingency tables (Chi-square or Fisher's exact test) or with the non-parametric Mann–Whitney-*U*-test depending on the data scale. The level of significance was set with *p* < 0.05.

## Results

Ten patients were included in this analysis and were treated with PROMOAT. The majority of the patients were males (90%). The mean age was 49.4 ± 15.9 years (95% CI: 60.4–71.1). The patients had a mean body mass index (BMI) of 26.2 ± 6.2 kg/m^2^ (95% CI: 24.7–28.8). These parameters did not differ between the two mesh groups.

**Figure 5:**
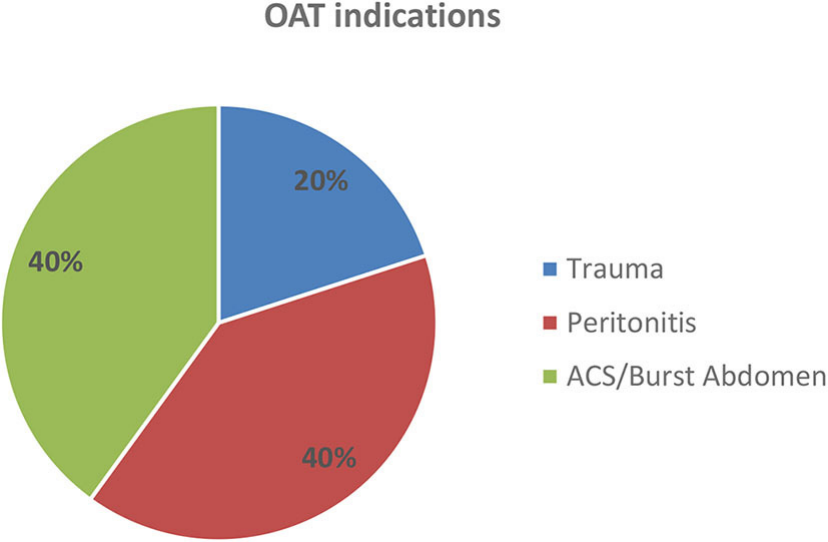
Indications for OAT. ACS, abdominal compartment syndrome.

The indications for OAT were trauma (2 cases, 20%), peritonitis (4 cases, 40%), and ACS or burst abdomen (4 cases, 40%) (Figure 5). There was no trauma among the long-term absorbable mesh patients. Underlying diagnoses are given in Table 1.

**Table 1:** Overview of diagnoses and OAT indications.

**Case no**.	**Diagnosis**	**OAT indication**
1	Severe sepsis (pneumonia)	ACS/burst abdomen
2	Abdominal aortic aneurysm repair (EVAR) with post-interventional bleeding	ACS/burst abdomen
3	Strangulated incisional hernia with sigmoid volvolus	Peritonitis
4	Serosal leak after sigmoid colectomy	Peritonitis
5	Severe sepsis (pneumonia)	ACS/burst abdomen
6	Sigmoid diverticulitis with free perforation	Peritonitis
7	Motor vehicle accident with blunt abdominal trauma and gastric perforation	Trauma
8	Motor vehicle accident with blunt abdominal trauma and hepatic and splenic laceration	Trauma
9	Capillary leak syndrome after urinary bladder resection (urothelial cell carcinoma)	ACS/burst abdomen
10	Intraabdominal abscess following colonic perforation	Peritonitis

*EVAR, endovascular aortic aneurysm repair; ACS, abdominal compartment syndrome*.

OAT duration was 21.0 ± 12.6 days (95% CI: 16.9–25.1). Definitive fascial closure was achieved in all cases. In 5 cases (case no. 1–5; 50%), a long-term absorbable alloplastic mesh was implanted and a non-absorbable alloplastic mesh in the remaining cases (case no. 6–10; 50%). OAT duration was for the long-term absorbable mesh group 28.3 ± 11.9 days (95% CI: 22.4–34.1) and for the non-absorbable mesh group 19.2 ± 12.6 days (95% CI: 13.6–24.7). This difference was not statistically significant (*p* = 0.111).

**Figure 6:**
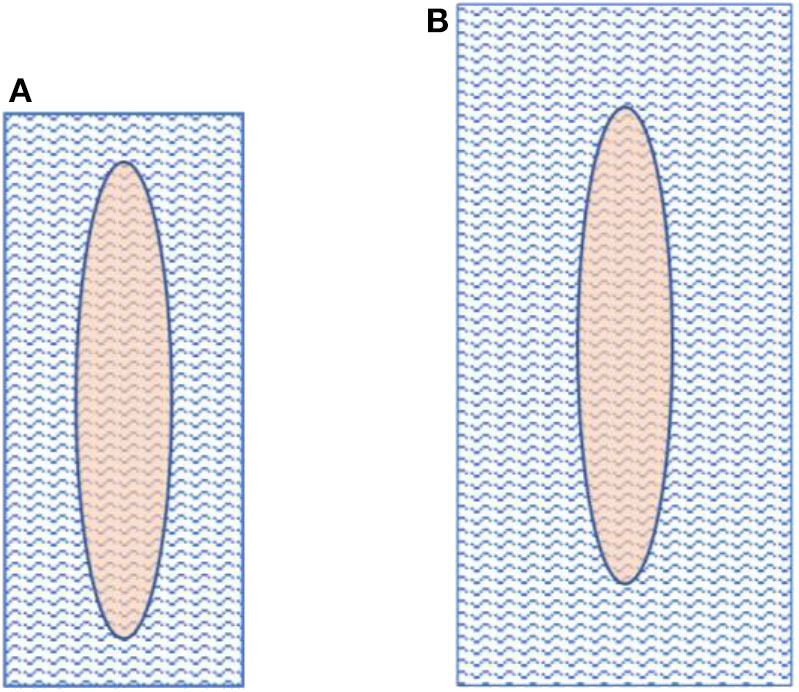
Fascial gap size in comparison to mesh size. The left image **(A)** depicts a mesh-to-gap ratio of 3 and the right one **(B)** a mesh-to-gap ratio of 5.

The mesh and fascial gap sizes are given in Table 2. The dimensions of the implanted meshes were 3- to 5 fold the sizes of the remaining fascial defect when definitive fascial closure was performed. Figure 6 visualizes the relations of mesh overlap.

**Table 2:** Comparison of study data across mesh groups.

	**All (n = 10)**	**Long-term absorbable mesh (*n* = 5)**	**Non-absorbable mesh (*n* = 5)**	**p**
Age [years]	49.6 ± 15.9 (95% CI: 44.4–54.8)	56.9 ± 13.4 (95% CI: 51.0–62.8)	43.7 ± 15.3 (95% CI: 37.1–50.4)	0.413
Sex (m/f)	9 (90%)/1 (10%)	5 (100%)/0 (0%)	4 (80%)/1 (20%)	0.556
BMI [kg/m^2^]	26.8 ± 6.2 (95% CI: 24.7–28.8)	30.4 ± 7.2 (95% CI: 27.2–33.5)	23.8 ± 3.1 (95% CI: 22.5–25.2)	0.286
Gap width [cm]	4.2 ± 1.4 (95% CI: 3.8–4.7)	5.3 ± 1.5 (95% CI: 4.5–6.0)	3.4 ± 0.6 (95% CI: 3.2–3.6)	0.111
Gap length [cm]	18.4 ± 4.3 (95% CI: 17.0–19.8)	19.1 ± 4.2 (95% CI: 17.1–21.2)	17.8 ± 4.1 (95% CI: 16.0–19.6)	0.905
Gap area [cm^2^]	81.1 ± 42.3 (95% CI: 67.2–94.9)	105.8 ± 48.4 (95% CI: 82.0–129.5)	61.3 ± 20.6 (95% CI: 52.3–70.3)	0.286
Mesh width [cm]	9.5 ± 2.2 (95% CI: 8.8–10.2)	9.4 ± 3.3 (95% CI: 7.8–11.0)	9.6 ± 0.8 (95% CI: 9.3–9.9)	0.286
Mesh length [cm]	29.1 ± 3.4 (95% CI: 28.0–30.2)	28.8 ± 1.3 (95% CI: 28.1–29.4)	29.4 ± 4.1 (95% CI: 27.6–31.2)	1.000
Mesh area [cm^2^]	277.6 ± 76.5 (95% CI: 252.6–302.6)	272.4 ± 105.3 (95% CI: 220.8–324.0)	281.8 ± 39.9 (95% CI: 264.3–299.3)	0.556
Mesh/gap area ratio	4.3 ± 1.8 (95% CI: 3.7–4.9)	3.5 ± 2.2 (95% CI: 2.4–4.6)	4.9 ± 1.0 (95% CI: 4.5–5.4)	0.413
Incisional hernia	0%	0%	0%	1.000
Pain [NRS]	2.3 ± 1.4 (95% CI: 1.9–2.8)	1.8 ± 1.8 (95% CI: 0.9–2.6)	2.8 ± 0.8 (95% CI: 2.4–3.2)	0.556
Complications	3 (30%)	1 (20%; infected seroma)	2 (40%; superficial SSI)	0.655

The follow-up interval was in mean 12.4 ± 10.8 months (range 2–30 months). The long-term absorbable mesh group had a longer follow-up period (23.5 ± 6.3 months, range 15–30 months) as this mesh type was implanted at the beginning of the study. The non-absorbable mesh group was followed up after 3.6 ± 1.0 months (range 2–5). During the follow-up period, complications were reported in three cases (30%). All of them were classified as Clavien-Dindo grade II (complications requiring non-surgical/pharmacological treatment). In one case, the complication was an infected seroma (long-term resorbable mesh), and in two cases, it was a superficial surgical site infection (both non-absorbable mesh group). None of the complications required invasive or surgical treatment. Apart from this, no other complications, especially no incisional hernias, were present.

At the follow-up exam, the overall pain was rated for all patients 2.3 ± 1.4 (95% CI: 1.9–2.8), for long-term resorbable meshes 1.8 ± 1.8 (95% CI: 0.9–2.6) and for non-absorbable meshes 2.8 ± 0.8 days (95% CI: 2.4–3.2). At rest pain was rated for all 1.0 ± 1.2 (95% CI: 0.6–1.4), for long-term absorbable meshes 1.8 ± 1.3 (95% CI: 1.1–2.4) and for non-absorbable meshes 0.4 ± 0.5 (95% CI: 0.2–0.6). Under strain all patients rated the pain 4.2 ± 1.0 (95% CI: 3.4–4.6), the patients with the long-term absorbable meshes rated 4.0 ± 1.2 (95% CI: 3.4–4.6) and patients with the non-absorbable mesh rated 4.4 ± 0.8 (95% CI: 4.1–4.7). None of the differences were statistically significant.

## Discussion

The results of this cohort study are promising. They might support the hypothesis that prophylactic onlay mesh implantation is feasible and safe after OAT, irrespective of whether a long-term absorbable or a non-absorbable mesh is used. During the follow-up period, no incisional hernias were observed, and the occurred complications in both groups were of minor concern and healed without invasive measures (Clavien-Dindo II).

Numerous studies evaluated the effect of prophylactic mesh implantation after laparotomies, and their results favored the prophylactic mesh implantation in high-risk patients over suture-only fascial closure (14, 15). For example, Jairam et al. conducted a randomized controlled trial (PRIMA trial) and found an incisional hernia incidence of 13% for the prophylactic onlay mesh compared to 31% for suture-only (14). The included patients had risk factors like aortic aneurysms and obesity. Seromas occurred in approximately one-quarter of the cases in the onlay mesh group. As in our study, those seromas had no impact on reoperations, invasive treatment, or surgical site infections. Muysoms et al. reported similar results in aortic aneurysm patients and prophylactic sublay mesh implantation (24).

In line with the findings for prophylactic onlay and sublay mesh implantation, Kohler et al. reported a reduced incisional hernia rate for prophylactic IPOM (intraperitoneal onlay mesh) implantation (7.2%) compared to suture-only (18.5%) (25). Patients in the IPOM group complained of more post-operative pain and had a longer duration of wound healing, however. For a similar technique, prophylactic implantation of a 7.5 cm wide IPOM stripe were incisional hernia rates of 17% after 2 years and 26% after a 5 year period reported (26, 27).

Borab et al. calculated an incisional hernia risk reduction of 85% in high-risk patients with elective laparotomies and prophylactic mesh implantation in a systematic review (15). Moreover, they found an increased rate of post-operative seromas, especially for onlay position of the mesh and polypropylene material, and more post-operative pain compared to suture-only fascial closure. Likewise, a meta-analysis by Indrakusuma et al. found a substantial incisional hernia risk reduction in aortic aneurysms repair patients and prophylactic mesh implantation (28). They reported no difference in the reoperation rate (i.e., due to hernia repair later on) between prophylactic mesh and suture-only groups, though. Concerning this study, Wanhainen emphasized there is level-A evidence for the prophylactic mesh implantation in open abdominal aortic aneurysm repair. Still, yet this is not represented in treatment guidelines, and it is not common in the daily routine (29). Wanhainen supposed most surgeons are hesitant to implant prophylactic meshes as long-term data is still lacking, and there might be only little individual experience in prophylactic mesh implantation. There are only a few studies with quite a long follow-up interval of about 5 years. However, these studies did not report any severe or frequent complications following prophylactic onlay mesh implantation (27, 30).

Eventually, there are several well-designed studies that support the beneficial role of prophylactic mesh implantation in high-risk patients with elective laparotomies (14, 15, 25, 28, 29). This will likely be reflected in upcoming updates of guidelines on abdominal surgery. However, small bites technique, a suture length to wound length ratio of >4:1 with a long-term or non-absorbable running suture is still the current state of the art of abdominal wall closure following the European Hernia Society guidelines of the abdominal wall closure (9, 11, 17).

Less research has yet been done on prophylactic mesh implantation in emergency laparotomies. But this is an important aspect, as the midline laparotomy is usually the first-choice abdominal incision in the emergency situation. Alternative methods like minimally invasive procedures with their inherently reduced incisional hernia risk, are hardly feasible. Burns et al. recently published a meta-analysis with 299 pooled patients and found substantially reduced incisional hernia risks and no remarkable differences concerning post-operative complications (31).

Put together; we hypothesized that the high-risk conditions in terms of incisional hernia development are similar or even worse in OAT patients (11). Firstly, OAT patients are equally likely to have intrinsic or patient-specific risk factors like aortic aneurysm or obesity. Secondly, the index operation at the initiation of OAT is usually an emergency operation. Thirdly, the repetitive traumatization of the abdominal fascia, caused by multiple reoperations and fascial traction, serves as a particular risk factor for incisional hernia development. And lastly, several pathophysiological factors (e.g., extended ICU stay, hemodynamic instability, malnutrition, catabolic nutritional status, and prolonged immobilization) are very likely to impair fascial viability, healing capabilities, and long-term resistance against hernia development. Hence, the pathophysiological conditions of elective or emergency laparotomies, for which the impact of prophylactic mesh implantation has already been studied, can be compared only to a limited degree. Nevertheless, as the incisional hernia rates after OAT are high, we supposed a positive impact of PROMOAT on incisional hernia rate (11).

Guidelines by the European Hernia Society on the fascial closure recommend mesh reinforcement in OAT or burst abdomen at the definitive fascial closure operation to reduce the incisional hernia rate (9). This recommendation was given based on weak evidence, as there were only very heterogeneous case series available. However, the guideline authors conducted a pooled analysis and found an incisional hernia rate of 19.4%. That was a substantial reduction compared to reported incisional hernia rates of 35–66% after OAT (9). Moreover, the pooled analysis revealed a rate of surgical site occurrences (surgical site infections, hematomas, and seromas) of 31.9%, which was higher in comparison to closure techniques without the use of mesh. Finally, the expert panel concluded there was expert guidance for mesh reinforcement at the definitive facial closure operation after OAT. The individual decision is up to the surgeon, though, in the context of increased risk for surgical site occurrences. (9, 11)

Only two studies were found, which prospectively evaluated prophylactic mesh implantation in OAT patients. The first one was published by Jakob et al. and evaluated the VAC-IPOM technique (32). With this, an IPOM is used for fascial traction in the VAWCM concept. Complete fascia-to-fascia closure was not mandatory, as the IPOM was considered stable even if there was residual fascia dehiscence at the end of OAT. Eventually, they reported a fascial closure rate of only 26% in the VAC-IPOM group compared to 74% in the VAWCM group. Nevertheless, in the VAC-IPOM group, longer hernia-free survival, and fewer reoperation were observed. The rate of post-operative wound infections was substantially higher in the VAC-IPOM group.

In our opinion, a prophylactic alloplastic IPOM should not be the treatment of the first choice since anatomical, functional abdominal wall reconstruction is advisable. Furthermore, IPOM should be implanted with caution due to the risk of an acute or chronic mesh infection, if potentially infectious intrabdominal foci are evident. Therefore, the implantation of a mesh in onlay position at the end of OAT (PROMOAT) is considered safe, as control of the infectious disease is then usually achieved. We would also suppose, the onlay mesh implantation is technically more straightforward than the IPOM implantation in an early stage of OAT (11, 14). But it has to be considered that there is currently no evidence to conclude on the appropriate mesh position (onlay, sublay, IPOM) for prophylactic implantation after OAT (11).

The second study on prophylactic mesh implantation in OAT patients was published by Petersson et al. (33). This report described a novel technique; the vacuum-assisted wound closure and permanent onlay mesh mediated fascial traction (VAWCPOM) for temporary and final closure of the open abdomen. The main difference to conventional VAWCM technique lies in the fact that the mesh, which is used for fascial traction during OAT, is placed in onlay position, is left there, and readapted in the midline with a suture when definitive fascial closure is performed. Moreover, the fascial edges are previously reinforced using a non-absorbable suture (reinforced tension line). At the end of OAT, the definitive fascial closure is obtained with a running suture, and the previously implanted mesh augments the stitches and the fascial edges. The authors reported an incisional hernia rate of 22.2% after a mean follow-up of 467 days and only minor complications without the need for invasive treatment.

The study by Petersson et al. describes a technically similar concept of augmenting the abdominal fascia with a prophylactic onlay mesh after OAT. Moreover, they found no substantial complications and a low incisional hernia rate. These results are in line with our findings. The higher incisional hernia rate of 22.2 vs. 0% in our study should be interpreted with caution, as our follow-up period is somewhat shorter, and incisional hernias are known to occur not necessarily shortly after definitive fascial closure. Probably, the midline incision and suturing of the prophylactic mesh might impair the mechanical properties, which might explain the higher incisional hernia rate by Petersson et al.

We suppose two further factors are of importance with regard to the prophylactic onlay mesh implantation. Firstly, as alloplastic meshes were used, we would favor implantation only in clean wound conditions with definitively controlled intraabdominal septic foci. And secondly, the mandatory NPWT of at least two changes after the onlay mesh implantation seems necessary, as seromas are a common and potentially infective complication following onlay mesh implantation. Our data showed only one seroma (11.1%), which is quite a low rate compared to other studies (14–16).

The question of which mesh material should be used for prophylactic onlay mesh implantation cannot be answered based on the scarcity of published data (11). Our study findings suggest there is no difference between long-term absorbable and non-absorbable mesh material. Still, of course, the power of this small case series is not sufficient, and long-term data are lacking to conclude on that. Hence, the choice of the mesh material should be made upon the surgeon's experiences. The current evidence on mesh materials confirms alloplastic non-absorbable meshes to be safe for prophylactic implantation (10, 11).

Study limitations comprise of the low power due to the small sample size. That hampers the possible conclusions drawn from this case series results. Moreover, there were differences between the mesh groups in the demographics and some operative variables (e.g., the gap width, mesh size). Though these differences were not statistically significant in this analysis, that is likely to be caused by the low sample size. Due to the short and unequal follow-up period, the reported outcome has to be interpreted with caution.

In conclusion, the prophylactic onlay mesh implantation of alloplastic, long-term absorbable, or non-absorbable meshes in OAT showed promising results and only a few complications that were of minor concern. Incisional hernias did not occur during follow-up. To validate the feasibility and safety of prophylactic onlay mesh implantation after OAT, long-term data and large-scaled prospective trials are needed.

## Data Availability Statement

The raw data supporting the conclusions of this article will be made available by the authors, without undue reservation.

## Ethics Statement

The studies involving human participants were reviewed and approved by Ethikkommission der Landesärztekammer Rheinland-Pfalz. The patients/participants provided their written informed consent to participate in this study.

## Author Contributions

All authors listed have made a substantial, direct and intellectual contribution to the work, and approved it for publication.

## Conflict of Interest

The authors declare that the research was conducted in the absence of any commercial or financial relationships that could be construed as a potential conflict of interest.
